# Effect of Preoperative Nerve Block on Postthyroidectomy Headache and Cervical Pain: A Randomized Prospective Study

**DOI:** 10.1155/2016/9785849

**Published:** 2016-03-13

**Authors:** Sunil Malla Bujar Barua, Anjali Mishra, Kamal Kishore, Saroj Kanta Mishra, Gyan Chand, Gaurav Agarwal, Amit Agarwal, Ashok Kumar Verma

**Affiliations:** ^1^GNRC Hospital, Guwahati, Assam 781030, India; ^2^Department of Endocrine Surgery, Sanjay Gandhi Postgraduate Institute of Medical Sciences, Raebareli Road, Lucknow 226014, India; ^3^Department of Anesthesia, Sanjay Gandhi Postgraduate Institute of Medical Sciences, Raebareli Road, Lucknow 226014, India

## Abstract

The aim of the current study was to investigate the efficacy of greater occipital nerve (GON) block and bilateral superficial cervical plexuses (BSCP) blocks in alleviating postoperative occipital headache and posterior neck pain after thyroidectomy. This randomized prospective study consisted of 75 women undergoing total thyroidectomy. Patients were randomized into three groups: Group I (*n* = 25): patients receiving GON, Group II (*n* = 25): patients receiving bilateral (BSCP) blocks, and Group III (*n* = 25): patients receiving no block. Assessment of occipital headache, posterior neck, and incision site pains was made at 12 hours and 24 hours after extubation by Visual Analogue Scale (VAS). In comparison to Group III significantly fewer patients in Groups I and II experienced occipital headache at 12 (*p* = 0.006) and 24 hours (*p* = 0.005) and also posterior neck pain at 24 hours (*p* = 0.003). Mean VAS scores at 12 and 24 hours for occipital headache (*p* = 0.003 and *p* = 0.041) and posterior neck pain (*p* = 0.015 and *p* = 0.008) were significantly lower in Group I. The differences between Groups II and III were not significant except for the occipital headache at 12 hours. The efficacy of GON block is superior to BSCP blocks in alleviating postthyroidectomy occipital headache and posterior cervical pain.

## 1. Introduction

It has been observed that many patients undergoing thyroidectomy develop postoperative posterior cervical pain and occipital headache in addition to the pain caused by the anterior neck incision. The reported prevalence of this problem is as high as 80% [1]. The effect of routine postoperative analgesics in alleviating headache and posterior neck pain is modest at best [1, 2]. Cervicogenic headache is a syndrome characterized by chronic hemicranial pain that is referred to the head from either bony structures or soft tissues of the neck. Sensory nerve fibers in the descending tract of the trigeminal nerve (trigeminal nucleus caudalis) are believed to interact with sensory fibers from the upper cervical roots. A functional convergence of sensorimotor fibers in the spinal accessory nerve and upper cervical nerve roots ultimately occurs with the descending tract of the trigeminal nerve and might also be responsible for the referral of cervical pain to the head [2–4]. Therefore, it is proposed that cervical spine structures innervated by the upper three cervical nerves (C1–C3) have the capacity to refer pain to the head and cause neck pain and headache. Hence, it can be presumed that various blocks directed at C1–C3 nerve routes should alleviate cervicogenic headache and pain in the neck [4].

The occipital nerve block has traditionally been used to diagnose and treat pain in the distribution of the occipital nerves (occipital neuralgia) and includes greater and lesser occipital nerves [5]. Greater occipital nerve (GON) block is considered to be an effective treatment in chronic headache and cervical pain [1, 6, 7]. The technique is associated with negligible complications. Han et al. found that preoperative GON block with 0.25% Bupivacaine was an effective technique in reducing occipital headache and posterior neck pain after thyroid surgery. While use of GON block for alleviating postthyroidectomy headache and neck pain has not been widely reported there are a number of reports on the effect of cervical plexus block for incision site pain following thyroidectomy [8–10]. In our practice we have found that patients who had undergone bilateral superficial cervical plexuses block (BSCP) tend to complain of less headache and neck pain in addition to incision site pain. Our colleagues in the department of anesthesia have been efficiently performing greater and lesser occipital nerve blocks for neurosurgical procedures. However, in our institute it has not been tried upon patients undergoing thyroidectomy. The primary aim of the current study was to investigate the efficacy of GON block and BSCP block in alleviating postoperative occipital headache and posterior neck pain after thyroidectomy.

## 2. Material and Methods

This prospective randomized study consisted of 75 females aged between 20 and 60 years undergoing total thyroidectomy with or without central compartment lymph node dissection in the department of endocrine surgery. Patients were randomized into three groups by envelope method: Group I: patients receiving GON block, Group II: patients receiving BSCP block, and Group III: patients receiving no block. An informed consent was taken from all. The number was calculated based on a pilot study revealing 70% prevalence of postthyroidectomy occipital headache and posterior neck pain in our patients. Based on this rate and accepted rate of 25% in treated group, and considering the power of 80% and significance of 0.05, the number calculated was 23 in each group. Two extra patients were enrolled to take care of any unforeseen exclusions. Hence, 25 patients were enrolled in each group. The initial plan was to use computer generated randomization sequence later; however, it was felt that for this small cohort even a small difference in number of patients in particular group might affect the significance of analysis; therefore, it was decided that envelope method of randomization shall be used to keep the numbers equal in all the groups. The institutional ethics committee approved the study protocol (IEC Code: 2012-137-MCH-64, Agenda Item number 14) and the study was registered with the Clinical Trials Registry India (CTRI/2013/01/003305; approved on 06/02/2013; principal investigator: Anjali Mishra).

## 3. Exclusion Criteria

Exclusion criteria are as follows:

1. patients undergoing modified radical neck dissection in addition to thyroidectomy,
2. patients undergoing endoscopic thyroidectomy,
3. patients with a recent or chronic history of headache or neck pain,
4. patients with a history of analgesic intake immediately prior to operation,
5. patients who were unable to understand the response grading of the Visual Analogue Scale [11],
6. a history of intolerance to the medications used in the study.

Informed consent was taken from all patients and detailed explanation about the procedure of nerve blockade and VAS evaluation was provided. Patients were asked to grade their pain on a scale of 1–10 with 0 meaning no pain and 10 meaning worst imagined pain VAS. A VAS score of more than 4 was considered as clinically significant pain. Patients were unaware of the group to which they were assigned to. Total thyroidectomy was performed under general anesthesia with endotracheal intubation. There was no deviation from the standard preanesthetic and anesthesia protocols and postoperative care remained unchanged. Patients were placed in the supine position on the operation table with the neck fully extended; a sandbag was placed below the shoulder as per standard departmental practice and thyroidectomy was performed by capsular dissection technique. All clinical details of the patient and response grading of the block were entered in a predesigned proforma.

### 3.1. Blocks and Anesthesia Protocol

**Figure 1:**
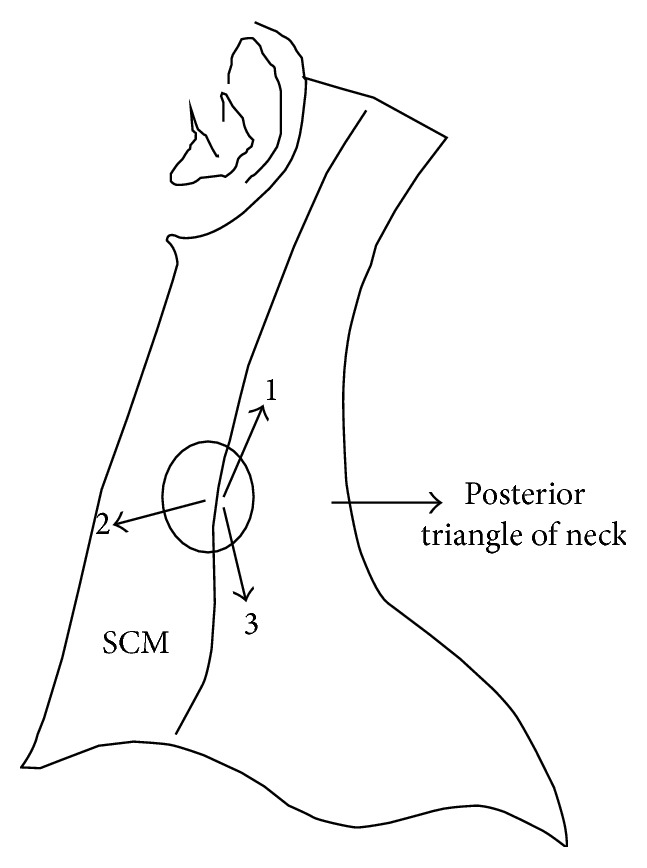
BSCP block is performed by tridirectional infiltration of 0.25% Bupivacaine. The point of insertion is the midpoint of the posterior border of the sternocleidomastoid (SCM). 2 mL each of Bupivacaine is infiltrated cranially (1) and caudally (3) and 1 mL horizontally over the muscle (2).

**Figure 2:**
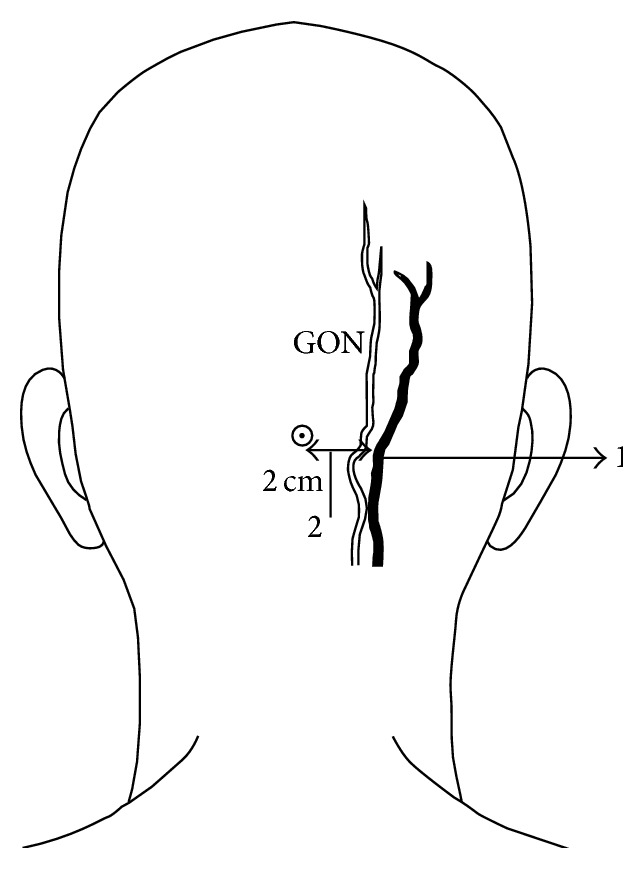
GON block is performed by infiltrating 5 mL of 0.25% Bupivacaine with needle inserted medial to the occipital artery (1). In cases where the artery is not palpable, the needle can be inserted to a depth of approximately 1 cm at a point 1 cm below and 2 cm lateral to the midpoint of the greater occipital protuberance (2).

Blocks were performed after induction of general anesthesia before positioning the patients for surgery by a consultant anesthesiologist. Premedication was performed with midazolam (0.05 mg/kg intravenously) 30 minutes prior to surgery. General anesthesia was induced with fentanyl (2 mcg/kg intravenously) and propofol (2 mg/kg intravenously). Following the administration of vecuronium (0.1 mg/kg intravenously), orotracheal intubation was performed. Anesthesia was maintained with sevoflurane 1-2 vol% in 50% O^2^/air with propofol 10–20 mL/hr in titrated doses. BSCP were performed in the supine position (Figure 1). A subcutaneous tridirectional infiltration of 5 mL of 0.25% Bupivacaine was performed bilaterally using a 22-gauge needle, the point of insertion being the midpoint of the posterior border of the sternocleidomastoid (2 mL each cranially and caudally and 1 mL horizontally over the muscle). Bilateral GON blocks were performed in the lateral position. Following slight flexion of the head, 5 mL of 0.25% Bupivacaine was infiltrated using a 22-gauge needle medial to the occipital artery. In cases where the artery was not palpable, the needle was inserted to a depth of approximately 1 cm at a point 1 cm below and 2 cm lateral to the midpoint of the greater occipital protuberance. Negative aspiration was performed prior to both blocks to prevent inadvertent intra-arterial injection (Figure 2).

Assessment of postoperative occipital headache, pain at the nape of the neck and incision site, and nausea and vomiting was made at 12 hours and 24 hours after extubation. The patients were provided VAS score sheets and they filled them independently. Outcome assessor was blinded to the group allocation of the patient. Rescue analgesia (diclofenac 75 mg) was given to the patient if the VAS score was more than 4. Same basic postoperative analgesics paracetamol (325 mg) and ibuprofen (400 mg) combination tablets three times a day were given to all the patients.

## 4. Statistical Analysis

An intent-to-treat analysis was conducted. Categorical variables were analyzed using the *χ* ^2^ test. The Kruskal-Wallis test was used to determine significant differences between groups for continuous variables with post hoc testing using the Mann-Whitney test. A *p* value of <0.05 was considered significant. Data was analyzed using SPSS version 15.0 (SPSS Inc., Chicago, IL).

## 5. Results

A total of 75 women with 25 women each in Group I, Group II, and Group III were included in this study. The mean age of the whole cohort was 39.8 ± 12.2 years (median 38 years). Of the 75 patients included in the study, 84% patients underwent total thyroidectomy alone while 16% underwent central compartment lymph node dissection. There is no significant difference in distribution of operative procedure between the groups (*p* = 0.743). The clinicopathologic attributes and other parameters relating to surgical procedure were also comparable among the three groups (Table 1). Mean hospital stay is 4 days in our study because as a protocol we do not discharge the patients unless their calcium levels are stable as the facility for monitoring serum calcium and dealing with symptomatic hypocalcemia is not adequate in our country. Postoperative temporary hypocalcemia occurred in 20, 24, and 24.0% of patients in Groups I, II, and III, respectively (*p* = 0.757), and one patient in Group I developed temporary recurrent laryngeal nerve palsy.

There was no morbidity related to either the BSCP or the GON in any patient. There was significant difference in proportion of patients reporting occipital headache at 12 and 24 hours, pain at nape of neck at 12 and 24 hours, and incision site pain at 12 hours between the blocked and unblocked groups (Table 2). The mean VAS scores at different sites also differed considerably between the groups (Table 3). The proportion of patients with VAS score more than 4 for occipital headache was also significantly different among the groups at 12 (0 versus 8.0 versus 20%; *p* = 0.049) but not at 24 hours (0 versus 8.0 versus 4.0%; 0.353). Also significantly fewer patients in Group I had VAS score more than 4 for the nape of neck pain at 12 (0 versus 20 versus 24.0; *p* = 0.037) but not at 24 hours (4.0 versus 12.0 versus 20.0%; *p* = 0.220). Almost similar findings were noted for incision site pain, that is, significantly less percentage of patients in Group I had VAS scores more than 4 at 12 hours (24.0 versus 40 versus 60%;  *p* = 0.035) but difference was not significant at 24 hours (40.0 versus 40.0 versus 44.0%; *p* = 0.946).

On separate analysis after excluding the 12 patients who underwent additional CCLND, the significance of the results remained the same. The VAS scores for occipital headache, incision site pain, and pain at the nape of the neck were significantly different between Groups I and III at 12 hours (*p* = 0.03, 0.06, and 0.07, resp.) and 24 hours (*p* = 0.04 and 0.035 and *p* ≤ 0.001, resp.). The VAS scores were also significantly different for occipital headache and pain at the nape of the neck at 12 hours (*p* = 0.044 and 0.044, resp.) and 24 hours (*p* = 0.023 and 0.031) between Groups II and III; however, the findings were not significant for incision site pain.

The percentage of patients fulfilling the criteria for rescue analgesia over and above the routine analgesics prescribed as per protocol was 28.0% in Group I, 64.0% in Group II, and 60.0% in Group III. This difference was significant (*p* = 0.02). The difference was still significant after excluding patients who underwent CCLND (Group 1: 7.9, Group II: 20.6, and Group III: 20.6%; *p* = 0.009). Postoperative nausea or vomiting at 12 and 24 hours, respectively, was noted in 4.0 and 0% in the greater occipital block group, 20.0 and 12.0% in the superficial cervical block group, and 12.0 and 0% in the unblocked group. This difference was significant at 24 hours (*p* = 0.044). Restriction of neck movements was reported by 80.0, 96.0, and 96.0% patients at 12 hours in Groups I, II, and III, respectively; the corresponding figures at 24 hours were 88.0, 96.0, and 96.0%, respectively. The difference was not significant (*p* = 0.080 and 0.424).

## 6. Discussion

Despite the high prevalence of postoperative headache and posterior neck pain after thyroidectomy, the majority of studies regarding postoperative analgesia have focused on incision site pain [1]. It is possible that the problem is more pronounced in patients with long standing large goiters like ours. In our study we found that both the occurrence and severity of occipital headache and posterior neck pain were less in groups receiving GON and bilateral BSCP blocks but the difference was significant only for the group receiving GON block. Though VAS scores were lower, the differences between Groups II and III were not significant except for the occipital headache at 12 hours. The relationship of GON, headache, and posterior neck pain and efficacy of GON blocks in alleviating these symptoms has been well documented in the literature [2–6]. During thyroidectomy the patient's neck is placed in a fully extended position. The proposed mechanism of postoperative headache and posterior neck pain is that the GON is vulnerable to injury during hyperextension of the neck and also it could get entrapped at potential entrapment points, that is, arch of C1 and lamina of C2 and the point at which it passes through the aponeurotic attachment of the trapezius and sternocleidomastoid muscle.

However, the efficacy of BSCB in reducing postthyroidectomy headache and posterior neck pain has not been documented previously. In our study we found reduction albeit nonsignificant in these pains. The possible explanation of pain reduction can be traced in the common origin and areas of distribution of GON and SCP. The GON and SCP share a common nerve route origin. The major part of the back of the skull though is supplied by the GON but the lateral part is supplied by a branch of the SCP (Lesser Occipital Nerve). Neck extension during thyroidectomy results in stretching of the sternocleidomastoid muscle which could compress the SCP at the point where these emerge from behind the sternocleidomastoid. Other explanations could be direct compression of the C2–C4 nerve roots between the scalenus anterior and scalenus medius muscles [12]. Besides, the spinal accessory nerve innervates the sternocleidomastoid and a functional convergence of sensorimotor fibers in the spinal accessory nerve and upper cervical nerve is known [2–4, 12]. Therefore, either direct compression of the SCP or the functional convergence of the spinal accessory nerve might be responsible for the pain referred to the head and the nape of neck. Hence, few proposed BSCP block for alleviating neck pain [4]. However, it has not been so far tried in reducing the severity of postthyroidectomy headache and posterior neck pain.

We had come across only one study using GON block for alleviating occipital headache and posterior neck pain after thyroidectomy. Han et al. in their study have shown that preoperative GON block with 0.25% Bupivacaine was effective in alleviating both of these symptoms [1]. BSCP block has traditionally been used to reduce incision site pain after thyroidectomy and its efficacy in achieving the said goal has been debatable. Some studies have shown a significant decrease in postthyroidectomy pain scores and analgesia requirements after BSCB whereas a few others reported that VAS scores and total patient controlled analgesia doses were comparable among groups of patients receiving or not receiving bilateral SCB [8–10]. The exact reason for the high incidence of postoperative nausea and vomiting in the BSCP block group in our study is difficult to explain and is contrary to some previous observations [13]. Emesis has multifactorial causation and it could not necessarily be correlated with a particular kind of block.

Performing BSCP blocks is far easier than GON blocks which require change in the position of the patient. If studies with larger sample size could show the effectiveness of BSCP block in reducing postoperative occipital headache and posterior neck pain. then BSCB could be recommended for the same. These aches and pains seem far more distressing to patients because while incision site pain can be easily tackled by increasing analgesics dosages, the same is not true about cervicogenic headache as is evident by its high prevalence in Group III. Another beneficial effect of applying blocks seems to be relief in restriction of neck movements.

The major limitation of this study is that the blocks were applied following induction of general anesthesia which precluded the assessment of the effectiveness of the block and it was an intent-to-treat analysis. Use of ultrasound would have ensured that the identified structures were successfully targeted or still better blocks could be performed prior to induction of general anesthesia. Further perception of pain is subjective and the varying pain tolerance in different populations is also well known. Therefore, other studies are required to validate the findings of the current study.

## 7. Conclusions

In conclusion, we found that the efficacy of GON is superior to BSCB blocks in alleviating postthyroidectomy occipital headache and posterior cervical pain.

## Figures and Tables

**Table 1:** Clinicopathologic profile of various groups.

Sl number	Attributes	Group I *n* = 25	Group II *n* = 25	Group III *n* = 25	Significance (*p* value)
1	Age: years (mean ± sd)	40.7 ± 12.2	38.1 ± 13.2	40.5 ± 11.3	0.714
2	Weight: kg (mean ± sd)	55.1 ± 8.7	62.1 ± 12.4	60.6 ± 10.7	0.100
3	Height: cm (mean ± sd)	154.2 ± 6.1	155.7 ± 6.7	156.5 ± 4.7	0.482
4	Goitre: *n* (%)				
	STN^*∗*^	04 (16.0)	04 (16.0)	03 (12.0)	
	MNG^†^	16 (64.0)	17 (68.0)	17 (68.0)	0.943
	Diffuse	04 (16.0)	03 (12.0)	05 (20.0)	
	Diffuse with nodule	01 (4.0)	01 (4.0)	— (0)	
5	Goiter weight: gm (mean ± sd)	121.8 ± 140.3	105.0 ± 77.1	140.2 ± 135.6	0.675
6	Duration of surgery: minutes^‡^ (mean ± sd)	255.6 ± 66.5	253.8 ± 72.2	242.0 ± 46.1	0.965
7	Postoperative stay: days (mean ± sd)	4.5 ± 1.0	4.6 ± 1.3	4.0 ± 1.0	0.06

^*∗*^STN: solitary thyroid nodule, ^†^MNG: multinodular goiter. ^‡^From the time of induction to extubation.

**Table 2:** Outcome in terms of occurrence of postoperative pain.

Sl number		Group I *n* = 25	Group II *n* = 25	Group III *n* = 25	Significance (*p* value)
1	Occipital headache (%)				
	12 hours	12 (48.0)	13 (52.0)	22 (88.0)	0.006^*∗*^
	24 hours	09 (36.0)	12 (48.0)	20 (80.0)	0.005^†^
2	Pain at nape of neck (%)				
	12 hours	14 (56.0)	16 (64.0)	21 (84.0)	0.092
	24 hours	12 (48.0)	17 (68.0)	23 (92.0)	0.003^‡^
3	Pain at incision site (%)				
	12 hours	23 (92.0)	24 (96.0)	25 (100.0)	0.353
	24 hours	25 (100.0)	24 (96.0)	24 (96.0)	0.598

^*∗*^I versus III, *p* = 0.006; II versus III, *p* = 0.014; and I versus II, *p* = 0.773; ^†^I versus III, *p* = 0.004; II versus III, *p* = 0.038; and I versus II, *p* = 0.567.

^‡^I versus III, *p* = 0.001; II versus III, *p* = 0.074; and I versus II, *p* = 0.252.

**Table 3:** Outcome in terms of VAS score.

Sl number		Group I *n* = 25	Group II *n* = 25	Group III *n* = 25	Significance (*p* value)
1	VAS at 12 hours: mean (median)				
	Incision site	2.6 ± 1.8 (3.0)	3.3 ± 2.1 (3.0)	4.3 ± 2.1 (4.0)	0.019^*∗*^
	Nape of neck	1.0 ± 1.0 (1.0)	1.7 ± 2.1 (1.0)	2.6 ± 2.3 (2.0)	0.015^†^
	Occipital headache	0.7 ± 0.9 (0.0)	1.2 ± 1.3 (1.0)	2.1 ± 1.7 (2.0)	0.003^‡^
2	VAS at 24 hours: mean (median)				
	Incision site	3.0 ± 1.5 (3.0)	3.4 ± 1.8 (3.0)	3.5 ± 1.8 (3.0)	0.510
	Nape of neck	1.0 ± 1.4 (0.0)	1.4 ± 1.4 (1.0)	2.1 ± 1.5 (2.0)	0.008^*δ*^
	Occipital headache	0.8 ± 1.1 (0.0)	1.0 ± 1.5 (0.0)	1.4 ± 1.1 (1.0)	0.041^§^

^*∗*^I versus III, *p* = 0.006; II versus III, *p* = 0.079; and I versus II, *p* = 0.316; ^†^I versus III, *p* = 0.004; II versus III, *p* = 0.091; and I versus II *p* = 0.287.

^‡^I versus III, *p* < 0.001; II versus III, *p* = 0.032; and I versus II, *p* = 0.33; ^*δ*^I versus III, *p* = 0.003; II versus III, *p* = 0.057; and I versus II *p* = 0.209.

^§^I versus III, *p* = 0.018; II versus III, *p* = 0.069; and I versus II, *p* = 0.515.
